# Liver Glycerol Permeability and Aquaporin-9 Are Dysregulated in a Murine Model of Non-Alcoholic Fatty Liver Disease

**DOI:** 10.1371/journal.pone.0078139

**Published:** 2013-10-30

**Authors:** Patrizia Gena, Maria Mastrodonato, Piero Portincasa, Elena Fanelli, Donatella Mentino, Amaia Rodríguez, Raúl A. Marinelli, Catherine Brenner, Gema Frühbeck, Maria Svelto, Giuseppe Calamita

**Affiliations:** 1 Department of Biosciences, Biotechnologies and Biopharmaceutics, University of Bari *Aldo Moro*, Bari, Italy; 2 Department of Biology, University of Bari *Aldo Moro*, Bari, Italy; 3 Department of Biomedical Sciences and Human Oncology, University of Bari *Aldo Moro*, Bari, Italy; 4 Metabolic Research Laboratory, Clínica Universidad de Navarra, The Spanish Biomedical Research Centre in Physiopathology of Obesity and Nutrition, Pamplona, Spain; 5 Instituto de Fisiología Experimental, Consejo Nacional de Investigaciones Científicas y Técnicas, Universidad Nacional de Rosario, Rosario, Argentina; 6 Institut National de la Santé et de la Recherche Médicale U769, LabEx LERMIT, Université Paris-Sud, Châtenay-Malabry, France; 7 Centro di Eccellenza di Genomica in campo Biomedico ed Agrario, Bari, Italy; 8 Network of Apulian Public Research Laboratories “WAFITECH”, Bari-Lecce, Italy; Wageningen University, The Netherlands

## Abstract

One form of liver steatosis, namely Non-Alcoholic Fatty Liver Disease (NAFLD), is a worrisome health problem worldwide characterized by intrahepatic triacylglycerol (TG) overaccumulation. NAFLD is a common feature of metabolic syndrome being often associated with obesity, dyslipidemia and diabetes and mostly closely linked to insulin resistance. The mechanism of NAFLD pathogenesis is object of intense investigation especially regarding complex systems ultimately resulting in excessive TG deposition in hepatocytes. However, scarce is the attention about the relevance of hepatic import of glycerol, the other primary source (as glycerol-3-phosphate) of increased TG in hepatocytes. Obese leptin-deficient (*ob/ob*) mice, an animal model of NAFLD, were used to evaluate the functional involvement of Aquaporin-9 (AQP9), the major pathway of liver glycerol entry, in hepatosteatosis. By RT-PCR and qPCR, the level of *Aqp9* mRNA in the liver of starved obese mice was comparable with the corresponding control lean littermates. By immunoblotting, the AQP9 protein at the hepatocyte sinusoidal plasma membrane of obese mice was markedly lower (33%) than lean mice, a finding fully confirmed by immunohistochemistry. By stopped-flow light scattering, the liver glycerol permeability of *ob/ob* mice was significantly lower (53%) than lean mice, a finding consistent with both the observed down-regulation of AQP9 protein and increased level of plasma glycerol characterizing obese mice. In summary, our results suggest implication of AQP9 in liver steatosis. The reduction of hepatocyte AQP9 and, consequently, glycerol permeability might be a defensive mechanism to counteract further fat infiltration in liver parenchyma.

## Introduction

Liver steatosis is characterized by ectopic accumulation of fat (primarily triacylglycerols, TG) in hepatocytes in response to metabolic, toxic and viral insults [1]. The most frequent form, namely the non-alcoholic fatty liver disease (NAFLD), affects subjects who do not abuse alcohol and is recognized as the leading cause of chronic liver disease in adults and children [2], [3], [4]. NAFLD has an estimated prevalence of 20–40% in Western countries [5] and is emerging as health problem also in family practice [6]. NAFLD is frequently associated with another harmful condition, the metabolic syndrome, which encompasses several abnormalities such as insulin resistance or established type 2 Diabetes, increased visceral adiposity, overweight/obesity, dyslipidemia and blood hypertension [7], characteristics commonly associated with increased cardiovascular risk. The current Western diet, high in saturated fats and fructose, plays a significant role [8]. The most worrisome form of NAFLD is the inflammatory-fibrogenic form, namely non-alcoholic steatohepatitis (NASH) which carries a higher risk of developing liver cirrhosis, and hepatocellular carcinoma [9].

Several studies have been recently focusing on the pathogenetic pathways leading to excess of TG in hepatocytes in NAFLD/NASH. Dysregulated hepatic fatty acid export, oxidation and desaturation and altered systemic and hepatic insulin sensitivity (insulin resistance) are among the main pathways in NAFLD pathogenesis (for a Review see [1]). Altered glycerol uptake by hepatocytes is also a major intersecting component, however, the underlying mechanism has begun to be understood only recently. In this respect, Aquaporin-9 (AQP9), an aquaporin membrane channel protein belonging to the subgroup of “*aquaglyceroporins*” [10], [11], [12], was proven to be the primary route for glycerol imported by hepatocytes from portal blood [13], [14]. Major functional significance was found for AQP9 in maximizing liver glycerol import during states requiring increased glucose production [14]. Relevance of AQP9 in facilitating liver uptake of glycerol for *de novo* synthesis of glucose during starvation had been hypothesized since several years [15], [16], [17], [18], [19], [20].

In rodents, AQP9 is mainly expressed in the liver, at the sinusoidal domain of hepatocyte plasma membrane [21], and, to a lower extent, in epididymis, *vas deferens*, epidermis and brain [17], [22], [23]. In humans, AQP9 has been also reported in omental and subcutaneous adipocytes [24]. Several lines of evidence indicate that AQP9 has a pivotal role in glycerol and TG homeostasis: *Aqp9* null mice have increased plasma glycerol and TG levels [17], a finding reflecting the reduction in liver glycerol permeability [14]. In rodents, hepatic AQP9 is repressed transcriptionally by insulin [15] whereas AQP9 increases in states of insulin resistance [16], [17]. Obese patients with type 2 diabetes have reduced expression of liver AQP9, an observation that has been interpreted as a compensatory mechanism aimed at contrasting further development of hyperglycemia [24], [25], [26].

Little is know, however, about liver AQP9 involvement into the pathogenesis of NAFLD/NASH [27]. The available information is limited to the evaluation of transcript and protein levels without functional data [28]. Hence, the aim of the present study was to examine the effect of hepatocellular steatosis on the expression, localization and regulation of hepatocyte AQP9 and glycerol permeability in obese leptin-deficient mice (This work has been presented as an abstract in *Journal of Diabetes* [1(suppl. 1): A147, 2009] at the 3^rd^ International Congress on “*Prediabetes and Metabolic Syndrome*” that was held in Nice (France) on April 1–4, 2009.), a monogenic animal model of NAFLD [29]. Functional involvement of AQP9 in liver steatosis is shown providing important insights into the knowledge of NAFLD pathogenesis, with a major translational value.

## Materials and Methods

### Animals

Seven week-old male C57BL/6J normal (lean) or C57BL/6J *Lep^ob^/Lep^ob^* (obese) mice (Charles River, Calco, Italy) were allowed free access to a standard laboratory rodent diet (Altromin-Rieper, Vandoies, Italy) and water *ad libitum*. After their arrival, animals were housed for one week in air-conditioned room (22°C) with 12/12 hours dark-light cycle before being fasted for 18 hours and sacrificed by cervical dislocation. The protocol was conducted according to the European Guidelines for the Care and Use of Laboratory Animals (Directive 86/609) and approved by the local committee for animal experimentation (Comitato Etico per la Sperimentazione Animale, Università degli Studi di Bari *Aldo Moro*).

### Analytical Procedures

Blood glucose levels were measured using an Accu-Check Sensor (Roche Diagnostics, Mannheim, Germany) and a drop of tail vein blood. For plasma glycerol analysis, blood samples were collected from the right cardiac ventricle into heparinized tubes and centrifuged for 10 min at 4,000×*g* to remove blood cells. Plasma glycerol concentrations were determined by using a colorimetric enzyme method following the manufacturer’s instructions (EnzyChrom™ Glycerol Assay Kit, BioAssay System, Hayward, CA). The plasma levels of total cholesterol, triacylglycerols, free fatty acids and alanine aminotransferase (ALT) activity were assessed using specific quantitation kits from Sigma Chemical Company (St. Louis, MO). Plasma insulin was assayed by an ELISA kit (Millipore, Billerica, MA) as recommended by the manufacturer.

### Semi-quantitative RT-PCR

Total RNA was extracted from the liver of sacrificed mice using *Tri Reagent Solution* (Ambion, Foster City, CA) according to the manufacturer’s instructions. Two micrograms of total RNA from each sample were reverse transcribed to cDNA using High-Capacity cDNA Reverse Transcription Kit (Applied Biosystems, Foster City, CA). Equal amounts of the resulting cDNA were used as templates for the subsequent PCR reactions performed to analyze the transcriptional expression of *Aqp9* by using AmpliTaq® DNA Polymerase (Applied Biosystems) and the primer pairs m*Aqp9*-1 (5′-AGATGCCTTCTGAGAAGGAC-3′) and m *Aqp9*-2 (5′-CACTACATGATGACGCTGAG-3′) (amplicon size: 893 bp). RT-PCR reactions were performed as previously reported and normalized against the β-actin expression [30].

### Preparation of Hepatocyte Sinusoidal Membrane Vesicles

Vesicles of hepatocyte sinusoidal plasma membrane were prepared as previously reported [14]. Briefly, mice livers were quickly removed after the sacrifice and homogenized with a Potter-Elvehjem homogenizer (15 strokes at 500 rpm) in an ice-cold *isolation medium* (220 mM mannitol, 70 mmol/L sucrose, 20 mmol/L Tris-HCl, 1 mmol/L EDTA and 5 mmol/L EGTA; pH 7.4), added with a cocktail of protease inhibitors (1 mM PMSF, 1 mmol/L leupeptin, 1 mmol/L pepstatin A). The homogenate was centrifuged at 500×*g* for 10 min and the pellet consisting of nuclei and unbroken cells discarded; the resulting supernatant was centrifuged at 8,000×*g* for 20 min. The related pellet containing mainly mitochondria was discarded whereas the supernatant was centrifuged at 10,000×*g* for 15 min. The resulting pellet enriched in the hepatocyte sinusoidal membrane vesicles was collected. All centrifugations were carried out at 4°C. All chemicals were purchased from Sigma Chemical Company.

### Immunoblotting Analysis

Hepatocyte sinusoidal membranes (30 µg of proteins) prepared as above were separated electrophoretically and submitted to immunoblotting using anti-ratAQP9 antibodies (1 µg/mL; Alpha Diagnostics International, San Antonio, TX) as previously reported [14]. The immunoreactive bands were quantified by densitometry using ImageJ software (NIH, Bethesda, MD). The density of each band was normalized against that of the housekeeping protein β-actin.

### Histochemistry and Immunohistochemistry

After the sacrifice, mouse livers were quickly removed and weighted. Samples of each specimen were fixed overnight by immersion with 4% paraformaldehyde (PFA) before being included in the appropriate resin. For the histological studies, liver samples were processed for embedding in Epoxy resin-Araldite (M) CY212 (TAAB, Aldermaston, England) as previously reported [31], [32]. Semi-thin sections (2 µm thick), obtained using an Ultratome III ultramicrotome (LKB, Bromma, Sweden), were stained with a toluidine blue-periodic acid-Schiff (PAS-TB). The morphometric and quantitative estimation of fat accumulation was carried out by an automated computerized methodology using SHAPE (Waveng, Bari, Italy), a software using a morphological and a chromatic operator to avoid overestimates in selecting lipid droplets [31]. The analysis was carried out using at least six digital micrographs (original magnification, 60×) from each sample specimen stained with toluidine blue-periodic acid-Schiff staining. The extent of lipid accumulation was calculated as mean ± S.E.M. of the percentage of the area occupied by the lipid droplets versus the total area of the hepatic section. For the immunoperoxidase light microscopy, PFA-fixed liver specimens were included in a hydrophilic resin (Technovit 8100, Heraeus-Kulzer, Wehrheim, Germany) as previously reported [30]. Before staining, semi-thin sections were incubated for 5 min at 37°C in 0.01% trypsin in 0.1% CaCl_2_ (pH 7.8). AQP9 was localized by the peroxidase-antiperoxidase (PAP) method. Endogenous peroxidase was blocked by 1% H_2_O_2_ for 10 min at room temperature. Sections were then incubated overnight at 4°C with the AQP9 antibody at a final concentration of 3 µg/mL in blocking buffer (1% normal goat serum in PBS). Successively, sections were treated for 1 h at room temperature with goat anti-rabbit immunoglobulin G (Sigma) diluted 1∶100 in blocking buffer and then incubated with peroxidase-antiperoxidase (Sigma) at a dilution of 1∶100 for 1 h at room temperature. Finally, the immunolabeling was revealed by incubation with 3,3'-diaminobenzidine/H_2_O_2_ medium for 10 min at room temperature. Negative controls were performed by omitting the primary antibody. Sections were observed using a Nikon Eclipse 600 photomicroscope equipped with a Nikon DMX 1200 camera (Nikon Instruments SpA, Calenzano, Italy).

### Stopped-flow Light Scattering Measurements of Glycerol and Water Permeability

The size of the sinusoidal membrane vesicles prepared as above was determined using a N5 Submicron Particle Size Analyzer (Beckman Coulter, Palo Alto, CA) and by transmission electron microscopy. The time course of vesicle volume change was followed from changes in scattered light intensity at 10°C at the wavelength of 530 nm by using a BioLogic MPS-200 stopped-flow reaction analyzer (BioLogic, Claix, France) that has a 1.6 ms dead time and 99% mixing efficiency in <1 ms. For the glycerol permeability measurements, light scattering experiments were performed as previously described by submitting the vesicles to a 150 mmol/L inwardly directed gradient of glycerol [14], [30]. The glycerol permeability coefficient (*P*_gly_; cm/s) was computed by using the equation:
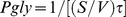
where S/V is surface-to-volume ratio and τ is the exponential time constant fitted to the vesicle swelling phase of light scattering time course corresponding to glycerol entry. For the water permeability measurement, aliquots of each membrane vesicle specimen were submitted to stopped-light scattering to evaluate the coefficient of osmotic water permeability (*P*_f_; cm/s). As previously described [30], vesicles were submitted to an inwardly directed hypertonic osmotic gradient of 140 mosM made by mannitol and the consequent vesicle shrinkage was followed as increase in scattered light intensity.

### Statistical Analysis

Experiments were performed at least in triplicate. All data resulted from four to six independent preparations were expressed as mean ± SEM. Data were analyzed statistically by the Student’s *t*-test. Results were considered statistically significant with a probability (*P*) of less than 5%.

## Results

### Evaluation of Hepatic Lipid Accumulation and Serum Parameters

**Figure 1:**
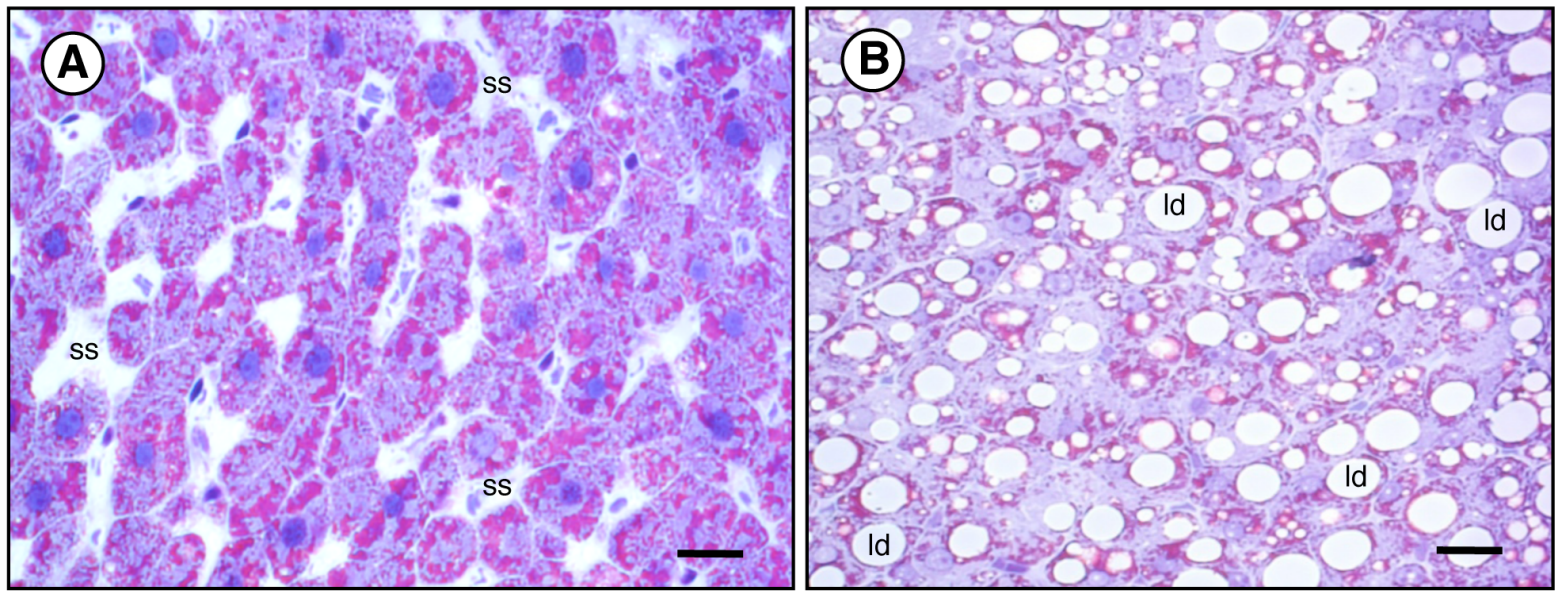
Histochemical features of liver parenchyma of C57BL/6J lean (A) and C57BL/6J *Lep^ob^/Lep^ob^* obese (B) mice. Semi-thin sections of the liver specimens were embedded in Epoxy resin and stained with Toluidine blue-PAS. Morphometric and semi-quantitative estimation of TG deposits in the liver parenchyma was assessed by an automated computerized methodology (see *Materials and Methods* for details). (**A**) Normal liver of C57BL/6J control lean mouse. (**B**) Simple steatosis with macrovesicular infiltration of hepatocytes in *ob/ob* mouse liver parenchyma. Hepatic parenchymal cells appear swollen with accumulated lipids which widen the parenchymal cell plates, narrow and distort the lumens of sinusoids so as to reduce the intrasinusoidal volume, as well as altering the architecture of the sinusoidal network. Many hepatocytes resemble mature adipocytes. *ld*, lipid droplet; *ss*, sinusoidal space. *Bar*, 30 µm.

Lipid accumulation in hepatocytes of obese and lean mice was estimated semi-quantitatively by histochemistry employing an automated computerized methodology previously devised [31]. Measurements are closely correlated with the intrahepatic accumulation of TGs. As expected, remarkable macrovacuolar liver steatosis was observed in the liver parenchyma of *Lep^ob^/Lep^ob^* mice whereas the surface area occupied by lipids in control lean mice had a physiological extent (43.3±5.2% *vs*. 3.1±1.6%, respectively) (Fig. 1A,B). Such histochemical pattern was consistent with increased body and liver weights and results of plasma gluco-lipidic parameters and ALT of *ob/ob* mice (Table 1).

**Table 1:** Metabolic plasma parameters in starved lean and *ob/ob* mice.

	Lean control	*ob/ob*
n	6	6
Body weight, grams	21.36±1.2	39.79±4.8*
Liver weight, grams	0.98±0.2	2.72±0.4*
Glucose, mmol/liter	8.33±0.8	17.92±3.2*
Glycerol, mmol/liter	0.62±0.1	0.96±0.1*
Triacylglyceroles, mmol/liter	0.56±0.1	0.82±0.1*
Free fatty acids, mmol/liter	0.49±0.1	0.71±0.1
Total cholesterol, mmol/liter	1.74±0.2	3.64±0.6*
Insulin, nmol/liter	0.31±0.1	9.61±0.3*
ALT, IU/liter	36.4±2.9	242.3±22*

ALT, alanine aminotransferase. Values are means ± S.E.M.; *n*, number of mice; *,*P*<0.01 lean compared with *ob/ob*.

### Hepatocyte AQP9 is Reduced in Liver Steatosis

**Figure 2:**
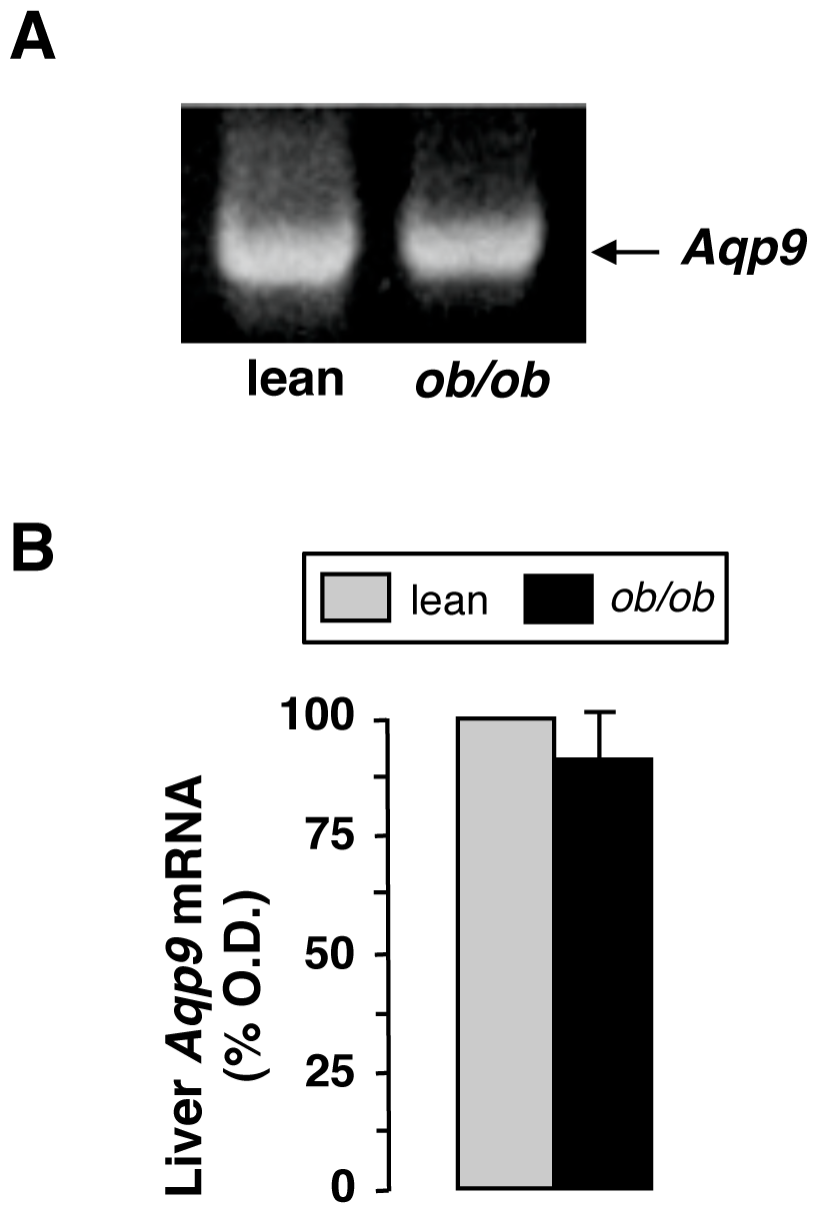
Expression analysis of liver *Aqp9* mRNA. (**A**) RT-PCR analysis, representative gel. The intensity of the *Aqp9* band (893 bp) is not significantly different between obese and lean mice. (**B**) Densitometric analysis of hepatic *Aqp9* mRNA expression normalized against that of the housekeeping gene ß-actin. The expression of *Aqp9* in lean mice is arbitrarily assumed as 100%. Data are mean ± S.E.M. (*n*, 6).

Expression of liver AQP9 in obese and lean mice was analyzed both at protein and mRNA levels. By RT-PCR, no significant changes were seen regarding the hepatic levels of *Aqp9* transcript between obese and lean mice (Fig. 2A,B). This result was confirmed by real time PCR (data not shown). No changes were also seen between obese mice and control lean littermates regarding the mRNA levels of *Aqp3* and *Aqp7*, the other known mouse aquaglyceroporins (AQP10, the fourth mammalian aquaglyceroporin, is a pseudogene in mouse). Actually, the hepatic levels of AQP3 and AQP7 transcripts remained steadily low (data not shown).

**Figure 3:**
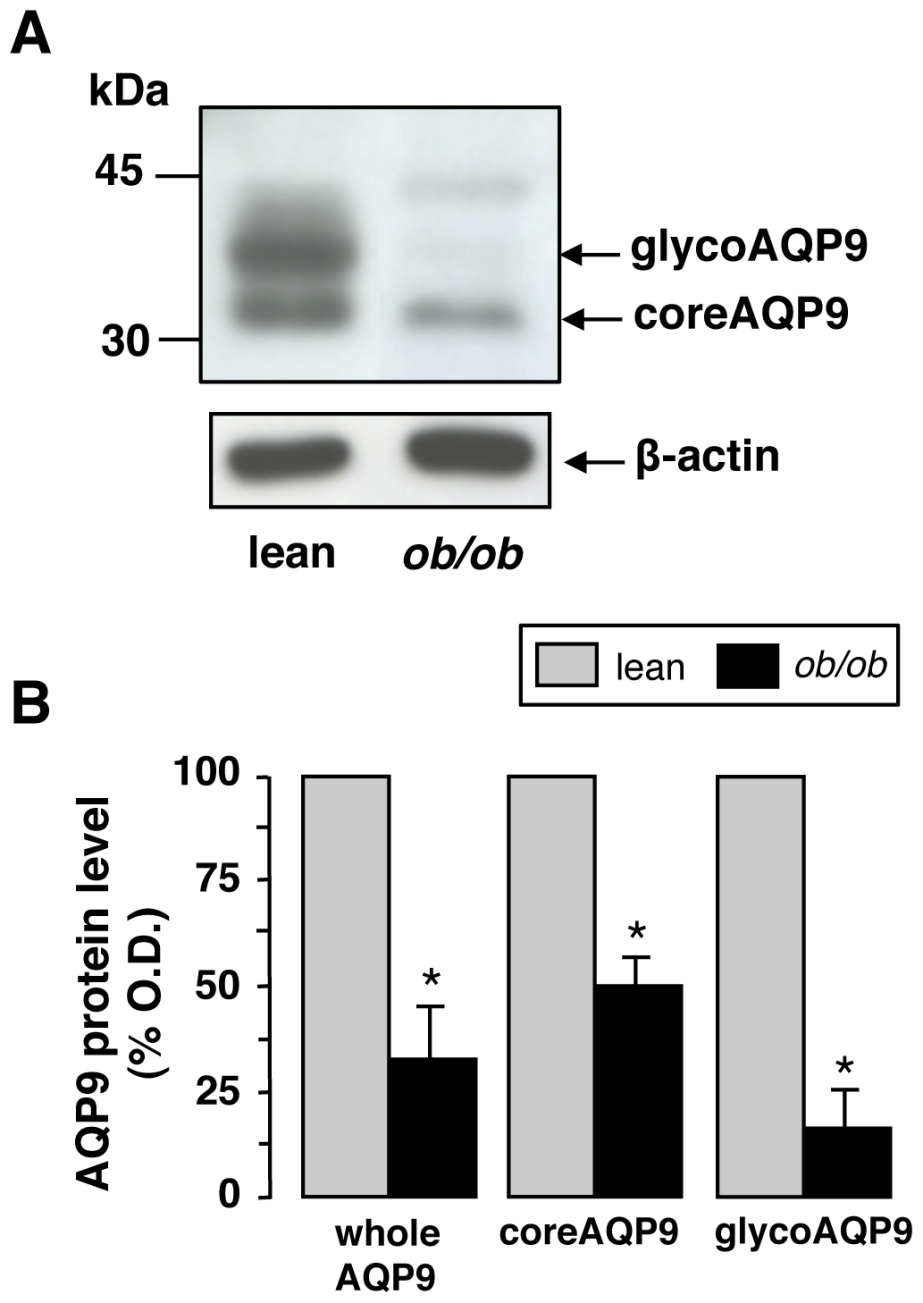
Immunoblotting analysis of hepatocyte AQP9. Immunoblots were carried out using the sinusoidal fraction of hepatocyte plasma membrane prepared by gravitational approach from the livers of obese mice and lean littermates as described in *Materials and Methods*. (**A**) Representative immunoblot for AQP9 normalized against the expression of the housekeeping protein ß-actin. Immunoreactive bands of 32 and 37–43 kDa, corresponding to the core (coreAQP9) and glycosylated (glycoAQP9) forms of AQP9, respectively, are detected. (**B**) Densitometric analysis of AQP9 immunoreactivity. Between the two forms of AQP9, the glycosylated one is that undergoing the strongest reduction. The expression of whole AQP9 (coreAQP9 plus glycoAQP9) in lean mice is arbitrarily assumed as 100%. Data are mean ± S.E.M. (*n*, 6). *P < 0.01.

**Figure 4:**
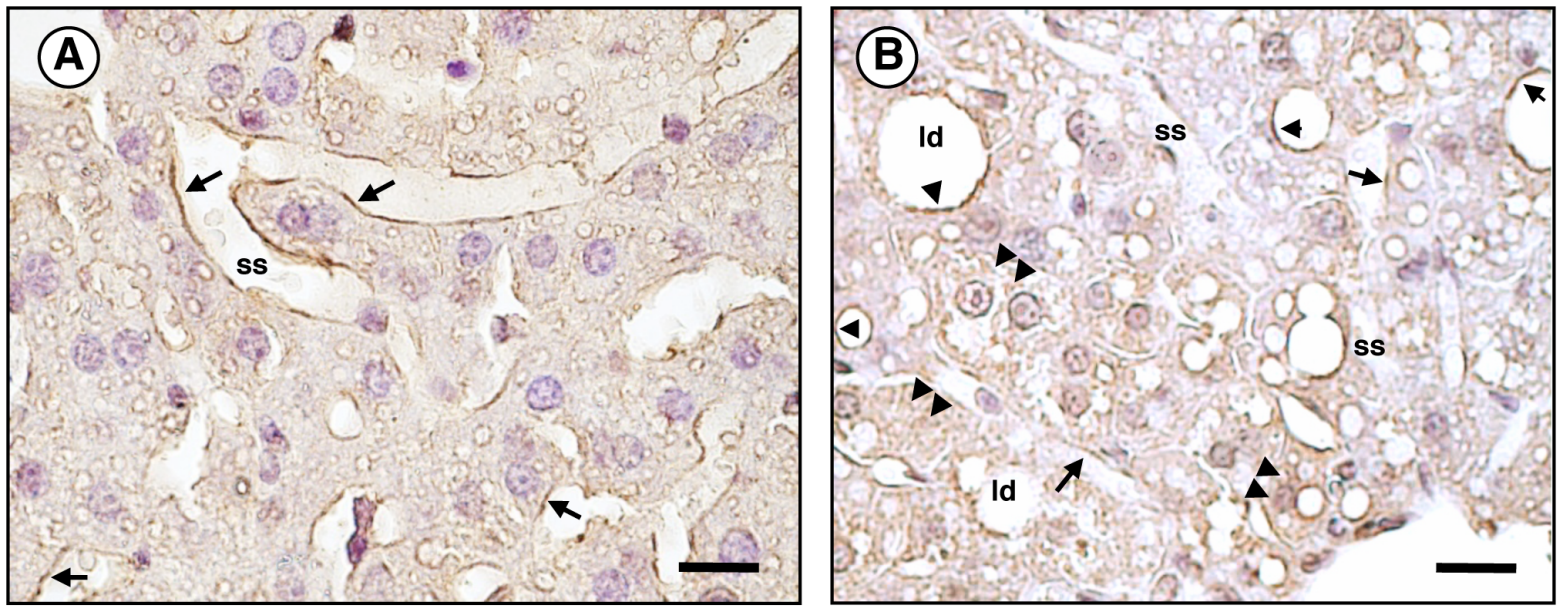
Immunohistochemical localization of liver AQP9 in *ob/ob* mice. Semi-thin liver sections submitted to immunoperoxidase as described in *Materials and Methods*. (**A**) Liver section from C57BL/6J control lean mouse. Immunolabeling (*brown staining*) is seen over the sinusoidal domain of the basolateral membrane of hepatocytes (*arrows; inset*). (**B**) Liver of C57BL/6J obese mice. Low AQP9 reactivity is present over the basolateral membrane of hepatocyte (*arrows*). Labeling is rather observed over the intracellular compartment (*double arrowheads*; *inset*). Large lipid droplets (*ld*), clear sign of macrovesicular steatosis, are seen in most hepatocytes. Considerable immunoreactivity is seen around the lipid droplets (*single arrowheads*) where organelles abound within the rim of cytoplasm surrounding the vesicle. *ss*, sinusoidal space. *Bar*, 30 µm.

The AQP9 protein was evaluated by semi-quantitative immunoblotting using hepatocyte sinusoidal plasma membranes prepared from mice after 18 hours of fasting, a condition maximizing the expression of AQP9 [14]. Immunoreactive bands of 32 and 37–43 kDa, corresponding to the core and N-glycosylated forms of the protein, respectively, were observed (Fig. 3A). A marked reduction (67%) of the whole AQP9 signal was seen in the *Lep^ob^/Lep^ob^* obese mice compared with C57BL/6J lean mice (Fig. 3A,B), a finding suggesting post-translation down-regulation. Notably, the extent of the decrease was much higher at level of glycosylated than core form of the protein (83% *vs*. 51%, respectively; *P*<0.01) (Fig. 3B). By immunohistochemistry, AQP9 was also significantly decreased at the sinusoidal domain of the hepatocyte basolateral plasma membrane of obese mice compared to lean mice (Fig. 4A,B). Results were in line with the immunoblotting studies. Increased AQP9 immunolabeling was observed in the hepatocyte intracellular compartment of *Lep^ob^/Lep^ob^* mice (Fig. 4B; *double arrowheads*). Immunoreactivity was particularly evident around the lipid droplets (*single arrowheads*) where, due to the macrovesicular steatosis, abundant organelles (e.g., rough and smooth endoplasmic reticulum, mitochondria) are usually localized in the attenuated rim of cytoplasm surrounding the lipid vesicle. No labeling was observed in negative control sections where the anti-AQP9 antibody was omitted (not shown).

### Steatosis is Associated to a Decrease in Liver Glycerol and Water Permeability

**Figure 5:**
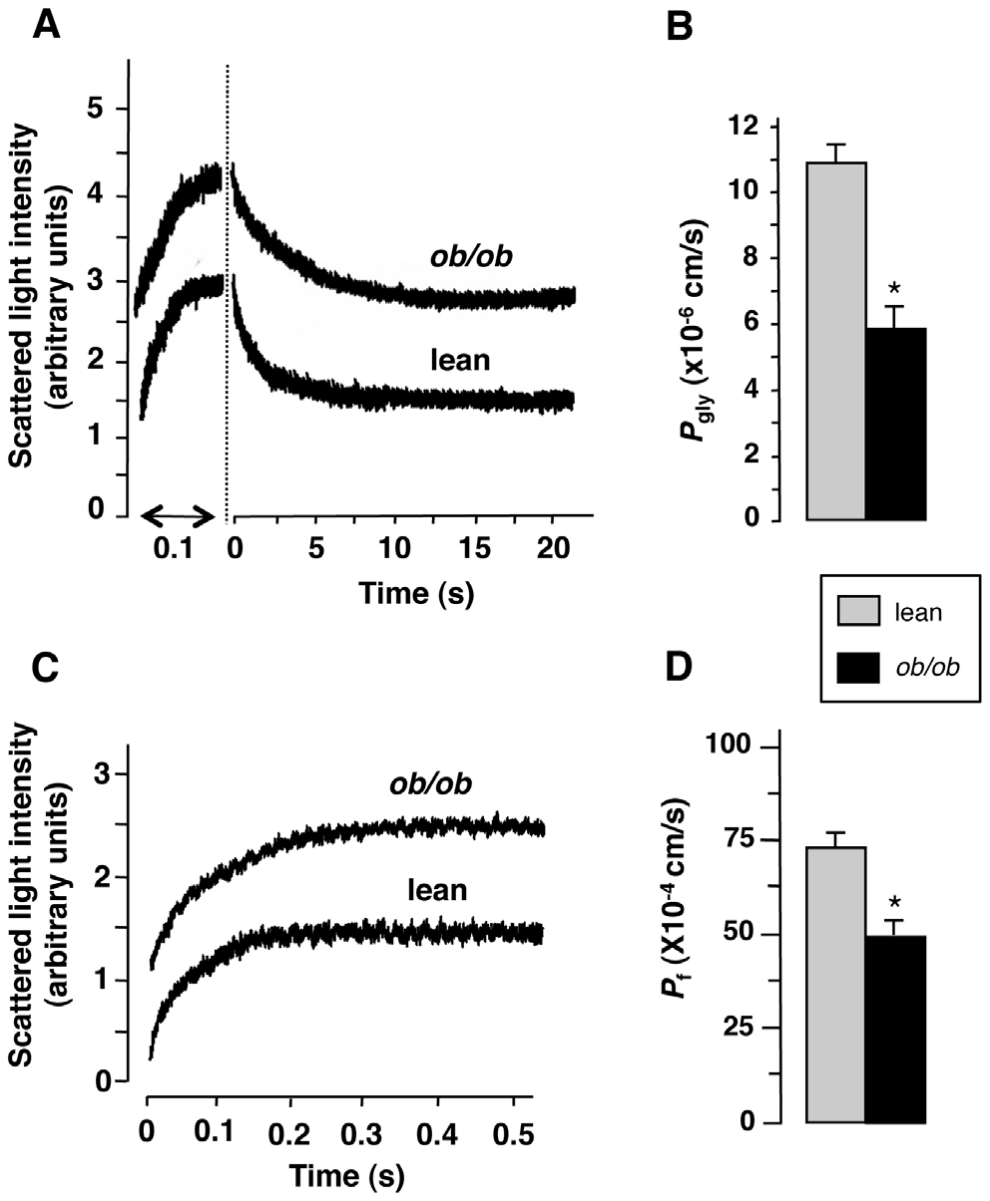
Liver glycerol and water permeability of obese and lean mice. The coefficient of membrane glycerol or water permeability (*P*_gly_ and *P*_f_, respectively) of the hepatocyte sinusoidal plasma membrane were assessed as described in *Materials and Methods*. (**A**) Glycerol permeability analysis. Representative tracings of stopped-flow light scattering of sinusoidal membrane vesicles from obese (*ob/ob*) and lean mouse livers in response to a 150 mM inwardly directed gradient of glycerol at 10°C. The initial increase in light scattering results from osmotic water efflux (vesicle shrinkage), followed by a slower decrease caused by glycerol entry. (**B**) The hepatocyte *P*_gly_ of *ob/ob* mice is significantly lower than lean littermates. (**C**) Osmotic water permeability analysis. Representative tracings of stopped-flow light scattering of sinusoidal membrane vesicles from *ob/ob* and control wild type mouse livers in response to a 140 mM inwardly directed hyperosmotic gradient of mannitol at 10°C. (**D**) The hepatocyte *P*_f_ of obese mice is moderately lower than that of lean mice. Data are mean ± S.E.M. from four independent experiments carried out using vesicles from each one of the liver preparations from six obese and six lean mice. *, *P*<0.01.

AQP9 is the primary route whereby glycerol is imported by mouse hepatocytes. Thus, we checked whether the down-regulation of AQP9 observed in obese mice was also associated with a reduction of the hepatocyte membrane glycerol permeability. Vesicles of sinusoidal domain of hepatocyte plasma membrane were prepared and the related coefficient of glycerol permeability (*P*_gly_; cm/s) measured by stopped-flow light scattering, as previously reported [14]. Consistent with both the reduction of sinusoidal AQP9 and increase in plasma glycerol concentration, the liver *P*_gly_ of the *Lep^ob^/Lep^ob^* mice was significantly lower than that of C57BL/6J lean mice (5.86±0.8 *vs*. 10.91±0.6×10^−6^ cm/s, respectively, at 10°C; Fig. 5A,B).

Because AQP9 channel allows also passage of water, we verified whether the steatosis-associated down-regulation of AQP9 was also accompanied by a reduction of osmotic water transport in hepatocyte sinusoidal plasma membrane. Water permeability was compared in sinusoidal membrane vesicles from livers of obese or control lean mice. The coefficient of osmotic water permeability (*P*_f_; cm/s) was measured by stopped-flow light scattering, as previously described [30]. Experiments were performed at 10°C. Consistent with the decrease of sinusoidal AQP9 and glycerol permeability seen above, there was a significant reduction in water permeability of the sinusoidal membrane vesicles of *ob/ob vs*. lean mice (*P*_f_ values of 49.91±4.9 *vs*. 72.83±4.2×10^−4^ cm/s, respectively; *P*<0.01) (Fig. 5C,D).

## Discussion

Liver steatosis is a multi-factorial disease where abnormal TG accumulation in the hepatocytes can result from a number of distinct alterations. Much attention has been recently given to the metabolic form of liver steatosis, NAFLD/NASH. Synthesis of TGs requires esterification of one glycerol molecule with three fatty acids, and AQP9 plays a major role in glycerol import by mouse hepatocytes. The way AQP9 is handled in hepatosteatosis may vary depending on the origin, grade and histological pattern of steatosis. Moreover, AQP9 expression in the liver might influence and/or might be influenced by ongoing liver steatosis.

In this study, the leptin-deficient obese adult mice, a murine model of NAFLD, displayed ectopic TG accumulation beside AQP9 down-regulation and consequent reduction of liver glycerol and water permeability. The *ob/ob* adult mouse was chosen as a well established model of “simple” liver steatosis [29] with high translational value into the human condition of metabolic fatty liver. Indeed, *ob/ob* mice resemble more the model of early steatosis (in our case this is due to the relatively young age of mice) and not steatohepatitis, i.e. more advanced stage of inflammatory and fibrotic modifications, a condition that in *ob/ob* mice is reached with the exposure to lipopolysaccharide [33]. The decreased AQP9 expression might be a defensive cell reaction for the steatotic hepatocyte.

A valuable result of this work regards the way AQP9, an aquaglyceroporin not acutely regulated by vesicular trafficking, is altered in hepatosteatosis. The observation that hepatocyte AQP9 is slightly diminished in terms of transcript whereas it is strongly reduced as protein indicates post-translational down-regulation. Moreover, the lower glycerol and water permeability correlated with AQP9 expression suggests that such post-translational regulation allows the hepatocyte to survive to stress rather being the consequence of a damage causing the dysfunction of the protein. Notably, most of the AQP9 reduction occurs at level of glycosylated form (Fig. 3A,B). It is conceivable to hypothesize that AQP9 is misrouted in its trafficking to the Golgi apparatus, following its synthesis at the rough endoplasmic reticulum. Misrouted hepatocyte AQP9 associated with decreased water and glycerol membrane permeability has also been reported in a rat model of extra-hepatic cholestasis [30]. In line with this scenario, immunohistochemical studies confirmed that obese mice show most AQP9 reactivity at the intracellular compartment of hepatocytes (Fig. 4B), whereas the immunolabeling at the plasma membrane is markedly reduced. Indeed, low levels of glycosylated AQP9 might result from inefficiency of Golgi apparatus, since excessive lipid storage in hepatocytes have been reported to lead to organelle (i.e., endoplasmic reticulum, ER) stress and failure together with proteasome dysfunction [34]. The ER is the organelle responsible for the initial steps of *N*-glycosylation of secretory, lysosomal, and many integral membrane proteins, and it is well known that structural changes in glycoproteins trigger ER stress and represent an indication for liver damage [35]. Thus, it seems plausible that the reduction of total and glycosylated AQP9 may be reflecting an increase in ER stress associated to fatty liver. In this regard, the potential crosstalk between ER stress and AQP expression has been recently reported by Rojek and colleagues [36] showing that the deletion of AQP11 results in disrupted ER homeostasis and increased sensitivity to ER injury upon metabolic challenge in the liver.

Glycerol utilization by hepatocytes is rate-limited by the membrane permeation step [37] and hepatocyte AQP9 plays a major role in facilitating glycerol import as proved experimentally by blocking chemically the related channel [13] or evaluating the liver glycerol permeability of wild type *vs* AQP9 null mice [14]. It is therefore reasonable to hypothesize that post-translational modifications of AQP9 may represent a way to modulate the glycerol influx at the physiological and pathological level. AQP9 is not only a glycerol and water channel, but also a urea transporter in murine hepatocytes. AQP9 contributes to urea transport in the basolateral membrane of murine hepatocytes, given that transgenic *Aqp9*-knockout mice showed a 30% decrease in urea permeability [38]. Thus, despite the protective role of AQP9 against hepatic steatosis, the effect of a sustained reduction of AQP9 on the accumulation of ammonia in the liver cannot be discarded. Further work is warranted to decipher the exact mechanism governing the down-regulation of AQP9 in hepatic steatosis. An interesting aspect that could be evaluated with other models, animals or cells is the impact of ER stress on the expression and function of AQP9. The involvement of signaling pathways such as the unfolded protein response (UPR), a pathway evoked in the degradation of abnormal protein aggregates and damaged organelles, is also matter for future investigation.

The hepatic down-regulation observed in the *ob/ob* mice should be contextualized on the metabolic features expressed by such model, namely hyperphagia, obesity, slight hyperglycemia, hyperinsulinemia, insulin resistance, hyperlipidemia, and hepatocellular steatosis. Up-regulation has been described for AQP9 in cultured human hepatoma cells treated with oleic acid, a compound with steatogenic effect [39]. Lowering of hepatocyte AQP9 in *ob/ob* mice may be a compensatory mechanism whereby liver counteracts further triacylglycerol deposition within its parenchyma. Alternatively, mechanisms involved in intrahepatic transport of glycerol might be affected during ongoing steatosis and become defective with further evolution of damage. A reduction of hepatocyte AQP9 protein with no changes in transcript level was recently observed in *n*3-PUFA (ω3 polyunsaturated fatty acids)-depleted female rats, a model of metabolic syndrome displaying several features of the disease also including liver steatosis [20]. Surprisingly, the down-regulation appeared to be accompanied by an increase in liver [U-^14^C]glycerol uptake. However, this interesting observation was not verified by assessing the liver glycerol permeability of the *n*3-PUFA-depleted rats. The apparent discrepancy between decreased AQP9 and increased uptake of glycerol was explained as due to a raise in glycerol kinase activity resulting from the alteration of the intracellular metabolism. Pathophysiological relevance of AQP9 in NAFLD was also suggested in a recent study using rats fed a high-fat diet (HFD) [28]. Reduction of HFD-induced steatosis was observed after decreasing the expression of liver *Aqp9* by RNA interference. Again, no functional analysis was carried out to link the expression of liver AQP9 with the extent of glycerol membrane permeability. One possibility is that AQP9 increases during early steatosis whereas it decreases at a later stage when consistent and excessive steatosis has occurred. Further studies are needed to verify this provocative hypothesis by evaluating the time course of hepatocyte AQP9 expression and glycerol permeability during the pathogenesis of liver steatosis. The expression and subcellular localization of hepatocyte AQP9 in steatohepatitis need also to be assessed.

The adipose tissue is the most important source of plasma glycerol. Under lipolytic conditions, TGs are hydrolysed into free fatty acids and glycerol, which are released into the bloodstream [40]. AQP7 constitutes the major glycerol channel in adipocytes, but our group has recently shown that AQP9 also represents a novel additional pathway for the transport of glycerol in human adipocytes [24]. In the present study, leptin-deficient *ob/ob* mice showed increased circulating free fatty acids and glycerol, suggesting an increased lipolytic activity in these genetically obese mice. Obesity has been associated with an increased gene and protein expression of AQP9 in human visceral fat [24]. Thus, it seems plausible that AQP9, if also expressed in murine adipocytes, is also dysregulated in mouse adipose tissue under obesogenic conditions. Further studies are warranted in order to ascertain this question.

## Conclusions

In summary, *ob/ob* mice show reduced liver AQP9 associated to a decrease in glycerol (and water) permeability, a finding that may explain the increase in plasma glycerol characterizing such animal model. Pathophysiological implication of AQP9 in hepatosteatosis is reasonably suggested. Because an intracellular glycerol accumulation triggers the enzymatic activity of glycerol kinase (GK), that catalyses the conversion of glycerol to glycerol-3-phosphate, thereby accelerating TG synthesis and leading to a progressive fat accumulation, the decrease in hepatic AQP9 and glycerol permeability may also constitute a defensive mechanism against an enhanced glycerol accumulation in order to prevent the observed hepatosteatosis in leptin-deficient *ob/ob* mice. However, we cannot exclude that during liver steatosis hepatocyte AQP9 undergoes to diverse mechanisms of dysregulation depending on the origin, pattern and extent of ectopic TG accumulation. Further investigation is needed, especially considering the complexity of the disease. Besides being a new important player in metabolic homeostasis, AQP9 may prove a new target to treat a common feature of metabolic syndrome such as NAFLD.
